# A cross-sectional study on menstrual health difficulties and premenstrual symptomatology in adolescent girls with intellectual disability: a salutogenic public health perspective

**DOI:** 10.3389/fpubh.2026.1771244

**Published:** 2026-05-05

**Authors:** Anlin Jenisha, Arun Sugumaran, Meena Ramanathan, Lalithambigai Chellamuthu, Kiruthika Asokan, Shahityaa Narayanaswamy

**Affiliations:** 1 Department of Community Medicine, Mahatma Gandhi Medical College and Research Institute, Sri Balaji Vidyapeeth University, Puducherry, India; 2 School of Yoga Therapy, Institute of Salutogenesis and Complementary Medicine, Sri Balaji Vidyapeeth University, Puducherry, India; 3 Department of Community Medicine, Vinayaka Mission's Medical College, Vinayaka Missions Research Foundation, Deemed to be University (VMRF-DU), Karaikal, Puducherry, India; 4 Department of Psychiatry, Mahatma Gandhi Medical College and Research Institute, Sri Balaji Vidyapeeth University, Puducherry, India; 5 Department of Obstetrics and Gynecology, Mahatma Gandhi Medical College and Research Institute, Sri Balaji Vidyapeeth University, Puducherry, India

**Keywords:** health promotion, intellectual disability, menstruation, multidisciplinary care, premenstrual syndrome, salutogenesis, yoga therapy

## Abstract

**Background:**

Adolescent girls with Intellectual disability (ID) are amongst the most vulnerable and underserved populations with special menstrual health needs. The prevalence of menstrual difficulties and premenstrual symptoms among people with ID often goes unrecognized, is poorly communicated and poorly managed which in turn contributes to functional limitations, increased caregiver burden and reduced quality of life. There is a lack of evidence from India regarding multidimensional PMS patterns and integrated supportive care approaches for girls with ID.

**Objective:**

To assess menstrual health difficulties and premenstrual symptom patterns among adolescent girls with intellectual disability and to describe multidisciplinary supportive care recommendations within a salutogenic public health framework.

**Methods:**

A cross-sectional school-based survey was conducted among 52 adolescent girls with mild to moderate ID aged 10–19 years in selected special schools of Puducherry. Menstrual history and hygiene practices were assessed by a gynecologist, and premenstrual symptoms across psychological, physical, and behavioral domains were evaluated using a caregiver-reported PMS screening tool over three consecutive cycles. Symptom clustering was performed by multidisciplinary experts. Exploratory inferential analysis was conducted using appropriate statistical tests to examine associations between level of intellectual disability and key menstrual health variables.

**Results:**

Dysmenorrhea was reported by 94.2% of participants with severe pain of 67.3%. Nearly half of them experienced menstrual irregularities, and over 90% needed partial to complete caregiver assistance in menstrual hygiene. Premenstrual symptoms were common across the domains: mood variability, irritability, pain, aggression, hypersomnia, and school absenteeism. Exploratory analysis showed no significant differences in symptom domain scores across levels of intellectual disability; however, hygiene dependence was significantly higher among adolescents with moderate intellectual disability. All participants exhibited overlapping symptom clusters-warranting integrated multidisciplinary recommendations. Routine supportive care gynecological, psychiatric, and yoga-based strategies were given. Emphasis was also laid on non-pharmacological management, behavior regulation, and body-mind coping.

**Conclusion:**

Adolescent girls with ID face a high burden of menstrual and premenstrual difficulties across physical, psychological, and behavioral domains. A salutogenic, multidisciplinary approach to gynecological care, together with behavioral support and yoga-based interventions, provided a culturally feasible framework for addressing menstrual health needs within special-school settings. For longitudinal effectiveness, such integrated strategies need to be evaluated.

## 1 Introduction

Adolescent girls with intellectual disability (ID) constitute a uniquely vulnerable population with complex health needs that often remain marginalized within mainstream public-health programs. Intellectual disability is defined as a disorder marked by substantial impairments in intellectual functioning and adaptive behavior that arise before to the age of 22 (1). Although the biological process of menarche in ID girls is comparable to neurotypical peers, their capacity to comprehend menstrual cycles, practice personal hygiene, and verbalize pain or discomfort is significantly compromised due to cognitive and functional limitations (2, 3). Evidence from India and other low- and middle-income settings shows that girls with ID often experience high caregiver dependence, poor self-care skills, behavioral disturbances during menstruation, and school absenteeism which are the factors that collectively restrict their social participation and quality of life (3, 4). In addition, national menstrual health initiatives rarely address disability-specific barriers that results in inadequate access to tailored education, adapted infrastructure, and supportive care (5).

Menstrual health problems in intellectually disabled adolescents deepen beyond dependence for hygiene management and affect more girls with dysmenorrhea, irregular periods, heavy menstrual bleeding, and menstrual-associated functional disability. Pain associated with menstruation is mainly severe, inadequately expressed, and undertreated, usually taking the form of behavioral disturbances rather than self-reported pain (6, 7). Menstrual irregularities along with prolonged menses and menstrual events add to the challenges of functioning in daily life and result in an increased care-giver burden and absenteeism from school. These problems are further exacerbated by the low knowledge of care-givers and school teachers about menstrual function and appropriate management, thus translating into delayed and irregular management of this health dilemma (2). Menstrual health management in intellectually disabled girls, therefore, tends to occur in a reactive rather than an anticipatory and structured and supportive manner.

Premenstrual symptoms (PMS), including emotional fluctuations, irritability, aggression, withdrawal, sleep disturbances, and somatic symptomatology, are expected to be more evident in girls with ID, yet remain under-recognized and under-reported (8, 9). Caregivers often interpret behavioral changes as part of the disability rather than menstrual-related mood shifts, which delays appropriate management and increases caregiver strain (10–12). There are only limited studies in India that have systematically characterized PMS symptom clusters in adolescent girls with ID or integrated assessments by gynecology and child psychiatry, Yoga specialists. This gap restricts the development of need-based, multidisciplinary strategies for menstrual wellbeing in this underserved group.

The concept of salutogenesis, which emphasizes resources that promote health, resilience, and coping rather than disease, provides a useful framework for understanding menstrual health in this population (13, 14). Salutogenic approaches shift attention from disease and deficits to capabilities, resilience, and supportive environments (13). This is particularly relevant for adolescents with intellectual disability, who require structured, predictable, and empowering interventions that build on their strengths and functional capabilities. In this context, routine supportive care practices including caregiver assistance, behavioral regulation strategies, and school-based support can be viewed as general resistance resources that enable adolescents to cope with menstrual related challenges despite functional limitations. These resources may enhance the comprehensibility, manageability, and meaningfulness of menstrual experiences, thereby facilitating better adaptation. Additionally, yoga-based practices, as culturally acceptable and low-cost interventions, align with this framework by promoting body awareness, relaxation, and emotional regulation (15). Evidence from general adolescent populations suggests that yoga can reduce dysmenorrhea, improve emotional regulation, enhance behavioral stability, and support autonomic balance (16, 17). However, its real-world applicability for menstrual and premenstrual difficulties among girls with intellectual disability has not been adequately explored.

The Union Territory of Puducherry, India, has a growing network of multidisciplinary services for children with disabilities; however, structured menstrual health pathways remain limited. Within this context, existing caregiving systems and school-based support structures may function as enabling resources for menstrual health management. In this study, a salutogenic framework is used to interpret menstrual health not only in terms of symptom burden but also in relation to such coping resources, including caregiver support, structured routines, and institutional environments.

To contribute to this evidence base, the present study aimed to assess menstrual health difficulties and premenstrual symptom patterns among adolescent girls with intellectual disability and to describe the multidisciplinary supportive care provided as part of routine care within a salutogenic public health framework.

## 2 Materials and methods

### 2.1 Study design and population

A school-based, cross-sectional study was conducted in special schools located within the field practice area of a tertiary care teaching hospital in Puducherry, India. The study targeted adolescent girls aged 10–19 years with a documented diagnosis of intellectual disability who had attained menarche and were enrolled in the selected special schools. Eligible participants included adolescents with mild to moderate intellectual disability, as verified through school or clinical records, along with the availability of a primary caregiver capable of providing reliable menstrual history. Adolescent girls who had not attained menarche, those with severe or profound intellectual disability, or cases where caregivers were unwilling or unable to provide informed consent were excluded from the study.

### 2.2 Sample size and sampling method

Sample size was calculated using the single-proportion formula *n* = *4pq/d*^2^. Based on the study by Ravi et al., (18) which reported that 87.7% of adolescent girls experienced at least one menstrual problem, the prevalence (*p*) was taken as 87.7%, and *q* as 12.3%. With a 95% confidence level and an absolute precision of 9%, the minimum required sample size was calculated as 51.16, which was rounded up to 52 participants. The sampling frame comprised all five special schools catering to children with intellectual disability located within the field practice area of a tertiary care teaching hospital in Puducherry. To ensure representation from both geographical contexts, the schools were stratified by location into urban (*n* = 3) and rural (*n* = 2) categories, and one school from each stratum was selected using simple random sampling by the lottery method, resulting in the inclusion of two schools, one urban and one rural. Within the selected schools, complete enumeration was undertaken, and all eligible adolescent girls aged 10–19 years who had attained menarche were approached consecutively.

### 2.3 Study procedure

Each participant underwent a single, structured multidisciplinary assessment session conducted within the school premises. A gynecologist, in the presence of the primary caregiver, obtained a detailed menstrual history including age at menarche, cycle regularity, flow characteristics, dysmenorrhea, hygiene practices, and prior treatment history, using a structured proforma. Subsequently, a child psychiatrist assessed premenstrual symptoms through caregiver report and behavioral observation using a brief premenstrual symptom screening checklist adapted for adolescents with intellectual disability, with reference to symptom occurrence across recent three menstrual cycles.

Following clinical assessment, the gynecologist and child psychiatrist jointly summarized menstrual and premenstrual difficulties and classified participants into symptom clusters (pain-related, behavioral, psychological, or mixed). Based on the identified symptom cluster, yoga-based supportive care recommendations were formulated by experts from the Institute of Salutogenesis and Complementary Medicine (ISCM), SBV. An appropriate yoga module comprising breathing practices, relaxation techniques, simple asanas, and guided relaxation was prescribed. The yoga modules were formulated in alignment with established, copyrighted therapeutic yoga frameworks by the institute, including the “DIVYANGA YOGA: The CYTER Model” (16808/2018-CO/L) and “MYFSC – Making Yoga Fun for Special Children” (10719/2020-CO/L) (19). Brief demonstrations and instructions were provided to the adolescent and the caregiver or teacher when present including practical guidance on menstrual hygiene practices and menstrual health education appropriate to the adolescent's cognitive and functional abilities. In addition, a brief educational session was conducted for school authorities during the visit to sensitize them to the menstrual hygiene needs of adolescents with intellectual disability and to promote improvements in supportive infrastructure, including access to clean, private, and menstrual friendly sanitation facilities.

**Figure 1:**
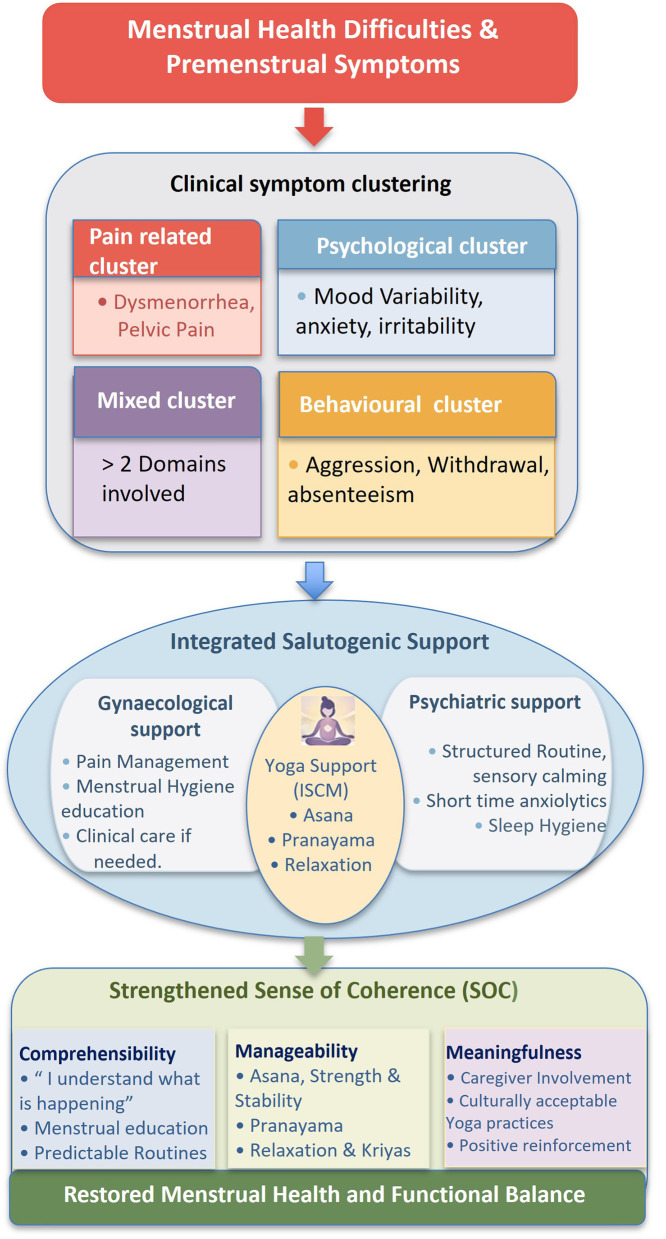
Salutogenic framework linking clinical symptom clusters to multidisciplinary supportive care for menstrual health among adolescent girls with intellectual disability.

These recommendations were documented as part of routine supportive care and were intended to enhance coping and self-regulation. From a salutogenic perspective, caregiver involvement, structured routines, and supportive school environments were considered as potential resources that may contribute to better comprehensibility, manageability, and meaningfulness of menstrual experiences in this population. The conceptual framework depicting this procedure is illustrated in Figure 1.

The study was informed by a salutogenic framework, which emphasizes health-promoting resources that support coping and functional adaptation. Although formal measurement of constructs such as Sense of Coherence (SOC) was not undertaken, elements related to General Resistance Resources (GRRs), including caregiver involvement, structured routines, and institutional support within school settings, were captured through clinical assessment, caregiver reports, and multidisciplinary observations. These elements were considered during interpretation of findings rather than as independently measured variables.

### 2.4 Data collection procedures

Data were collected through face-to-face caregiver interviews and structured clinical assessments conducted within the premises of the selected special schools. Data collection was carried out in a quiet, private room to ensure participant comfort and minimize behavioral distress among the adolescents. Following confirmation of eligibility and completion of informed consent and assent procedures, information was collected using structured proformas and screening tools administered by the multidisciplinary team.

The first section Captured Socio-demographic and disability-related details of the adolescent, including age, residence, level of intellectual disability, living arrangement, and co-morbid conditions, verified using caregiver reports and school or clinical records. The second section focused on menstrual health assessment and was administered by the gynecologist in the presence of the caregiver. This section documented age at menarche, cycle regularity and length, menstrual flow characteristics, dysmenorrhea and its severity, menstrual hygiene practices, problems experienced during menstruation, and any prior medical consultations or treatments. Findings were recorded using a structured menstrual health proforma.

The third section assessed premenstrual symptoms and behavioral changes using a caregiver-reported Premenstrual Symptoms Scale (CR-PMS), supplemented by behavioral observation. The CR-PMS was adapted from previously published instruments assessing premenstrual symptoms among adolescents and modified for caregiver-based reporting in adolescents with intellectual disability (20, 21).

The tool captures observable symptoms across psychological, physical, and behavioral domains using a five-point ordinal severity scale ranging from “not at all” to “severe.”

The instrument was used as a screening tool rather than a diagnostic tool. It underwent face and content validation by a multidisciplinary panel comprising experts in Community Medicine, Psychiatry, and Obstetrics and Gynecology. Pretesting was conducted among caregivers and teachers to ensure clarity, feasibility, and cultural appropriateness. Internal consistency reliability was assessed using Cronbach's alpha, which demonstrated acceptable reliability (α = 0.733). Caregivers reported symptom severity based on observations over the preceding three menstrual cycles, and domain-wise symptom patterns were subsequently derived for analysis.

Following clinical evaluation, participants were categorized into predefined symptom clusters, and the corresponding multidisciplinary recommendations were recorded in a standardized recommendation log. All completed forms were reviewed on-site for completeness and consistency on the day of assessment. Identifiable information was removed prior to data entry, and each participant was assigned a unique study identification number to maintain confidentiality.

### 2.5 Data analysis

Data were entered into Microsoft Excel and analyzed using IBM SPSS Statistics version 27 (IBM Corp., Armonk, NY, USA) (32). Descriptive statistics were used to summarize menstrual characteristics, premenstrual symptom patterns, symptom clusters, and multidisciplinary recommendations. Categorical variables were presented as frequencies and percentages, while ordinal and non-normally distributed variables were summarized using medians and interquartile ranges.

In addition, exploratory inferential analysis was performed. The Mann Whitney *U*-test was used to compare premenstrual symptom domain scores across levels of intellectual disability. Fisher Exact test was used to assess associations between categorical variables. A *p* value of less than 0.05 was considered statistically significant.

### 2.6 Ethical considerations

The study was conducted in accordance with the Declaration of Helsinki. Ethical approval was obtained from the Institutional Human Ethics Committee of MGMCRI, Sri Balaji Vidyapeeth University, Puducherry. (Ref no: MGMCRI/Res/01/2023/13/IHEC/12) Written informed consent was obtained from parents or primary caregivers, and assent was obtained from adolescents wherever feasible based on cognitive ability. Confidentiality and privacy were strictly maintained throughout the study.

## 3 Results

A total of 52 adolescent girls with intellectual disability were included in the study. The distribution of their sociodemographic and clinical characteristics is outlined. The majority of participants belonged to the late adolescent age group of 17–18 years, accounting for 71.2% of the study population, while 19.2% were aged 15–16 years. Only 9.6% of the participants were in the early adolescent age group of 12–14 years, indicating that most girls were in later adolescence. With regard to the level of intellectual disability, 67.3% of the adolescents had mild intellectual disability, while the remaining 32.7% were classified as having moderate intellectual disability. None of the participants were identified as having severe intellectual disability. In terms of place of residence, a slightly higher proportion of adolescents were from rural areas (55.8%) compared to those from urban settings (44.2%). Regarding living arrangements, 73.1% of the participants were living with their parents, whereas 26.9% were residing in hostels or institutional care facilities. With regard to medical co-morbidities, seizure disorders and thyroid disorders were the most commonly reported conditions, each affecting 13.5 % of the adolescents. Diabetes mellitus was present in 7.7% participants. Overall, 31 adolescents (59.6%) did not have any reported medical co-morbidities, indicating that a majority of participants were free from additional chronic health conditions apart from intellectual disability.

The distribution of menstrual characteristics and hygiene-related practices is shown in Table 1. Early onset of menstruation was common, with half of the participants (50.0%) attaining menarche before 12 years of age, while 36.5% experienced menarche between 12 and 14 years. Only a small proportion reported menarche after 14 years, indicating that most adolescents entered menstruation at an early age. With respect to menstrual cyclicity, 59.6% of girls reported regular cycles, whereas 40.4% experienced irregular cycles. Normal cycle length was observed in 46.2% of participants, while a substantial proportion reported prolonged cycles consistent with oligomenorrhoea. Shortened cycles suggestive of polymenorrhoea were noted in a smaller subset of adolescents.

**Table 1:** Menstrual characteristics and hygiene practices among adolescent girls with intellectual disability (*N* = 52).

Category	Number (*n*)	Percentage (%)
Age at menarche		
< 12 years	26	50.0
12–14 years	19	36.5
>14 years	7	13.5
Cycle regularity		
Regular	31	59.6
Irregular	21	40.4
Cycle length		
< 21 days (polymenorrhoea)	7	13.5
21–35 days (normal)	24	46.2
>35 days (oligomenorrhoea)	21	40.4
Dysmenorrhea		
Present	49	94.2
Absent	3	5.8
Severity of dysmenorrhea		
Mild	3	5.8
Moderate	14	26.9
Severe	35	67.3
Hygiene practices		
Independent	3	5.8
Partially dependent	21	40.4
Fully dependent	28	53.8
Problems during menstruation^*^		
Heavy flow	21	40.4
Clots	9	17.3
Pain	35	67.3
School absenteeism	14	26.9
Soiling accidents	14	26.9

^*^Multiple responses.

The study revealed a high burden of menstrual morbidity among the participants. Dysmenorrhea was reported by nearly all girls (94.2%), with severe pain being the predominant presentation, affecting more than two-thirds of the study population. Moderate dysmenorrhea was reported by 14 adolescents, while only three participants experienced mild symptoms. Menstrual hygiene practices indicated considerable dependence, as over half of the girls (53.8%) were fully dependent on caregivers, and 40.4% were partially dependent, with very few managing hygiene independently. Several menstrual-related problems were reported, with menstrual pain being the most common complaint, followed by heavy menstrual flow, affecting 21 girls. Functional consequences such as school absenteeism and soiling accidents were each reported by 14 participants, reflecting the impact of menstruation on daily activities and school participation. The presence of clots during menstruation was reported by 17.3% of the adolescents. These findings highlight the multifaceted nature of menstrual health challenges faced by adolescent girls with intellectual disability.

Premenstrual symptoms were assessed across psychological, physical, and behavioral domains (Table 2). In the psychological domain, mood variability was the most prominent symptom, with 30.0% reporting moderate symptoms, 16.7% relatively severe, and 33.3% experiencing severe fluctuations. Anger and irritability were also notable, with a combined 56.7% reporting relatively severe or severe symptoms. Poor concentration was prevalent, with 50.0% reporting mild symptoms and 36.7% experiencing moderate to severe difficulty. Depressed mood and anxiety affected a substantial proportion, with more than half reporting at least mild symptoms and the overall domain score was assessed to be median (IQR) of 1.67 (1.33–2.17).

**Table 2:** Domain-wise premenstrual symptom scores among adolescent girls with intellectual disability.

Domain	Symptom	Domain score median (IQR)
Psycholgical domain	Depressed mood	1.67 (1.33–2.17)
	Anxiety	
	Mood variability	
	Anger/irritability	
	Poor concentration	
	Decreased interest	
Physical domain	Breast swelling/tenderness	1.78 (1.56–1.89)
	Headache	
	Joint/muscle pain	
	Abdominal pain	
	Swelling of hands/feet	
	Frequent urination	
	Lower-back pain	
	Acne/skin changes	
	Nausea	
Behavioral domian	Increased appetite	1.81 (1.38–2.13)
	Food craving	
	Hypersomnia (excess sleep)	
	Difficulty sleeping	
	Avoidance of social contact	
	Prone to violence/aggression	
	School Abstinence	
	Personality change	

In the physical domain, several symptoms showed high severity levels. Joint or muscle pain and abdominal pain were predominantly in the moderate-to-severe range, affecting 80.0 and 73.3% of participants, respectively. Lower back pain was marked by high severity, with 53.3% reporting relatively severe pain and 16.7% reporting severe pain. Breast tenderness was moderate or higher among 86.7% of girls. In contrast, symptoms such as swelling of hands and feet and frequent urination were more commonly mild or not experienced and the domain score was derived to be Median (IQR) of 1.78 (1.56–1.89).

In the behavioral domain, a wide range of symptoms was reported across severity levels. School absenteeism during the premenstrual phase showed a striking concentration in the higher categories, with 63.3% reporting relatively severe and 26.7% reporting severe absenteeism. Avoidance of social contact was present in 33.3% with severe symptoms. Increased appetite, food craving, and hypersomnia were also frequent, with around one-third of participants experiencing moderate-to-severe symptoms. Aggressive behavior was moderate or worse in 70.0% of the sample. Personality changes were reported in varying severity, with 33.3% experiencing moderate symptoms and 20.0% experiencing relatively severe or severe alterations and the total domain score of median (IQR) is 1.81 (1.38–2.13).

Overall, the findings indicate that moderate to severe premenstrual symptoms were common across all domains, reflecting the heightened vulnerability of adolescent girls with intellectual disability to pronounced physical discomfort, mood changes, and behavioral dysregulation during the premenstrual period.

Gynecological recommendations were provided based on each adolescent's menstrual profile, symptom severity, and hygiene needs and is presented in Table 3. The majority of participants were advised non-pharmacological measures for menstrual pain management, with hot fomentation, adequate hydration, and rest recommended to 49 adolescents (94.2%). Hygiene-related counseling was universally provided, with all participants (100.0%) receiving advice on frequent pad changing, proper perineal hygiene, and safe disposal of used menstrual products.

**Table 3:** Gynecologist recommendations provided to participants.

Recommendation category	Type of recommendation	Specific advice	Participants advised *n* (%)^*^
Menstrual pain management	Non-pharmacological	Hot fomentation, hydration, rest.	49 (94.2)
Hygiene improvement	Non-pharmacological	Frequent pad change every 4 h, proper perineal hygiene by drying technique (wipe front to back), proper disposal of used menstrual products.	52 (100.0)
Diet and lifestyle	Non-pharmacological	Iron-rich diet, Omega 3 fatty acids, magnesium and potassium rich foods.	46 (88.5)
Symptomatic pain relief	Pharmacological	Analgesics/NSAIDS.	18 (34.6)
Anemia management	Pharmacological	Iron–folic acid along with deworming.	14 (26.9)
Clinical referral	Medical decision	PCOS, anemia, abnormal bleeding, thyroid disorders, eating disorders.	11 (21.2)

^*^Multiple recommendations were provided to the same participant based on clinical assessment; therefore, percentages do not total 100%.

Dietary and lifestyle recommendations, including encouragement of iron-rich foods and intake of omega-3 fatty acids, magnesium, and potassium-rich foods, were advised to 46 adolescents (88.5%). Pharmacological interventions were recommended selectively based on clinical need. Analgesics or non-steroidal anti-inflammatory drugs were advised to 18 participants (34.6%) for significant dysmenorrhea, while iron–folic acid supplementation with deworming was recommended to 14 adolescents (26.9%) where anemia was suspected.

Clinical referrals for further evaluation, including assessment for polycystic ovarian syndrome, anemia, abnormal uterine bleeding, thyroid disorders, or eating disorders, were advised for 11 participants (21.2%). These findings reflect a predominantly supportive, non-pharmacological approach with targeted medical management based on individual clinical profiles.

Psychiatric recommendations were provided based on behavioral and emotional difficulties identified during the assessment and is summarized in Table 4. Interventions targeting irritability and anger were most frequently advised, with structured routines, sensory-calming approaches, and caregiver cueing recommended for the majority of adolescents (47 participants, 90.4%). Behavior-modification techniques and positive reinforcement strategies were advised for aggression in nearly two-thirds of the sample (69.2%).

**Table 4:** Psychiatrist recommendations provided to participants.

Behavioral concern	Type of recommendation	Specific intervention	Participants advised *n* (%)^*^
Irritability/anger	Non-pharmacological	Structured routine, sensory calming, caregiver cueing	47 (90.4)
Aggression	Non-pharmacological	Behavior-modification techniques, positive reinforcement	36 (69.2)
Withdrawal/social disengagement	Non-pharmacological	Social reinforcement, structured engagement activities	29 (55.8)
Sleep disturbances/appetite changes	Non-pharmacological	Sleep hygiene, fixed bedtime schedule, regulated meals	34 (65.4)
Emotional dysregulation	Non-pharmacological	Supportive counseling, calming techniques	41 (78.8)
Severe behavioral concerns (if present)	Pharmacological^†^	Short-term anxiolytics/sleep-support medication	6 (11.5)

^*^Multiple recommendations were provided to the same participant based on clinical assessment; therefore, percentages do not total 100%. ^†^Pharmacological interventions were considered only in selected participants with severe behavioral concerns.

Social withdrawal and disengagement were addressed through social reinforcement and structured engagement activities in over half of the participants (55.8%). Sleep disturbances and appetite-related changes were common, and sleep-hygiene measures including fixed bedtime schedules and regulated meals were recommended for approximately two-thirds of the adolescents (65.4%). Supportive counseling and calming techniques for emotional dysregulation were advised in a substantial proportion of participants (78.8%), reflecting the high burden of affective and behavioral instability observed during the premenstrual period.

Pharmacological interventions were infrequently indicated and were reserved for adolescents presenting with severe behavioral concerns. Short-term anxiolytic or sleep-support medications were recommended in a small subset of participants (11.5%) following specialist psychiatric evaluation. Overall, the pattern of recommendations highlights a predominantly behavioral and supportive care approach, with pharmacological treatment used conservatively and only when clinically warranted.

The distribution of yoga therapy recommendations provided as part of routine supportive care is summarized in Table 5. All participants received a structured set of yoga-based recommendations tailored to address their predominant symptom patterns. A warm-up and preparatory module were commonly advised at the beginning of sessions, comprising quiet sitting, prayer, and play-way warm-up practices such as *Jathis* and *Kriyas*, typically lasting 5–10 min to facilitate physical readiness and attention.

**Table 5:** Yoga therapy recommendations.

Yoga module	Yogic techniques	Indicative duration and frequency	Participants advised *n* (%)^*^
Warm-up and preparatory module	Quiet sitting, prayer, play-way warm-up practices including Jathis and Kriyas	5–10 min at the beginning of each session	52 (100.0)
Asana module (psycho-physiological postures)	Tadasana, Trikonasana, Veerasana II, Meru Asana, Paschimottanasana, Bhujangasana, Setu Bandhasana, Pawanamuktasana, Eka/Dwi Pada Uttana Asana	10–15 min per session	46 (88.5)
Pain-relief and pelvic activation module	Gentle stretches and pelvic opening practices (Chatushpadasana, Baddhakonasana, Pawanamuktasana, Setu Bandhasana, Jathara Parivritti Asana)	10–15 min daily or on alternate days	44 (84.6)
Pranayama (mindful focused breathing) module	Mukha Bhastrika, Om Pranayama, Bhramari Pranayama	5–10 min daily	49 (94.2)
Relaxation and calming module	Shava Asana with Pranava Pranayama	5–10 min, preferably at the end of the session or before bedtime	50 (96.2)
Individualized integrated module	Combination of breathing, postures, and relaxation practices tailored to symptom cluster	As advised by the yoga therapist	31 (59.6)

^*^Multiple recommendations were provided to the same participant based on clinical assessment; therefore, percentages do not total 100%.

The asana module, consisting of psycho-physiological postures aimed at improving posture, flexibility, and pelvic circulation, formed a central component of the recommendations. This module included practices such as *Tadasana, Trikonasana, Veerasana II, Meru Asana, Paschimottanasana, Bhujangasana*, and *Setu Bandhasana*, and was generally advised for 10–15 min per session. For adolescents presenting predominantly with pain-related symptoms, a specific pain-relief and pelvic activation module was recommended, incorporating gentle stretches and pelvic opening practices to enhance comfort and circulation. These practices were advised for 10–15 min, either daily or on alternate days.

To address psychological and behavioral symptoms, a pranayama module focusing on mindful and calming breathing techniques such as *Mukha Bhastrika, Om Pranayama*, and *Bhramari Pranayama* was included, usually recommended for 5–10 min daily. In addition, a relaxation and calming module centered on *Shavasana* with *Pranava Pranayama* was commonly advised, particularly at the end of sessions or before bedtime, to promote relaxation and emotional regulation.

For adolescents presenting with mixed symptom clusters, an individualized integrated module was recommended. This module combined selected breathing practices, postures, and relaxation techniques, tailored to the individual's symptom profile, with duration and frequency advised on a case-by-case basis by the yoga therapist.

A comparison of premenstrual symptom (PMS) domain scores, menstrual cycle regularity, and hygiene dependence between adolescents with mild and moderate intellectual disability is presented in Table 6. The analysis showed no statistically significant differences in premenstrual symptom burden across the psychological (*p* = 0.936), physical (*p* = 0.460), and behavioral (*p* = 0.066) domains, indicating that the overall pattern and severity of symptoms were comparable between adolescents with mild and moderate intellectual disability.

**Table 6:** Association between level of intellectual disability and menstrual characteristics.

Variable	Mild ID (*n* = 35) *n* (%)	Moderate ID (*n* = 17) *n* (%)	*p*-value
PMS domain scores (median, IQR)			
Psychological	1.67 (1.29–2.50)	1.92 (1.29–2.17)	0.936^a^
Physical	1.78 (1.56–2.00)	1.67 (1.44–2.00)	0.460^a^
Behavioral	1.88 (1.50–2.13)	1.50 (1.22–2.00)	0.066^a^
Cycle regularity			
Regular	21 (60.0%)	10 (58.8%)	0.98^b^
Irregular	14 (40.0%)	7 (41.2%)	
Hygiene dependence			
Independent/partial	22 (62.9%)	2 (11.8%)	**0.007** ^b^ ^*^
Fully dependent	13 (37.1%)	15 (88.2%)	

Statistical tests performed:

^a^Mann–Whitney U-test;

^b^Fisher's Exact test;

^*^p < 0.05 considered statistically significant.

With respect to menstrual cycle regularity, a similar distribution was observed in both groups, with 60.0% of adolescents with mild intellectual disability and 58.8% of those with moderate intellectual disability reporting regular cycles (*p* = 0.98), indicating no association between level of intellectual disability and cycle pattern.

In contrast, a significant difference was observed in hygiene dependence. Among adolescents with mild intellectual disability, 62.9% were independent or partially dependent for menstrual hygiene, whereas only 11.8% of those with moderate intellectual disability demonstrated similar levels of independence. A markedly higher proportion of adolescents with moderate intellectual disability were fully dependent on caregivers (88.2%) compared to those with mild intellectual disability (37.1%), and this association was statistically significant (*p* = 0.007).

## 4 Discussion

This cross-sectional study examined the menstrual health profile, premenstrual symptoms, and functional limitations among intellectually disabled adolescent girls and identified the nature of gynecological, psychiatric, and yoga-based recommendations provided to their primary caregivers. The findings reveal a considerable burden of menstrual-related challenges in this population, consistent with earlier Indian and global literature reporting disproportionate vulnerability among adolescents with intellectual disabilities. At the same time, these findings also point toward the presence of potential coping resources within caregiving and institutional environments, which can be interpreted within a salutogenic framework.

In the present study, 94.2% of girls reported dysmenorrhea and two-thirds had severe pain. This prevalence is much higher than in general-population school studies, where dysmenorrhea is usually reported in 50%−70% of adolescents, with severe pain in about 10%−20% (22). Our results are more comparable to studies focused specifically on girls and women with intellectual disability, which consistently describe menstrual distress as frequent and functionally disabling. Nurkhairulnisa et al., (4) in a Malaysian study of girls with ID in institutional care, reported high rates of menstrual pain and associated distress, emphasizing the need for systematic menstrual disorder management in this group. Menstrual cycle irregularities were also common in our sample, with 40% reporting cycle length >35 days (oligomenorrhoea) and another 13.5% with polymenorrhoea. Similar patterns have been noted in disability cohorts, where comorbidities, psychotropic medications, antiepileptics, nutritional deficiencies and stress can affect cycle regularity (6, 23). In the present study, no statistically significant association was observed between cycle regularity and level of intellectual disability, suggesting that menstrual cycle characteristics may not differ substantially across functional categories.

These findings are further supported by clinical observations from the American Academy of Pediatrics, which highlight that adolescents with disabilities frequently present with dysmenorrhea, irregular bleeding, and premenstrual symptoms, particularly when families face challenges related to menstrual hygiene and behavioral management (7, 24). The systematic identification of these patterns through structured assessment offers an opportunity for anticipatory guidance, caregiver counseling, and regular clinical follow-up, thereby strengthening continuity of care.

### 4.1 Hygiene dependence and caregiver challenges

More than half of the girls in our study were fully dependent on caregivers for menstrual hygiene, and only a small proportion could manage pad changing and perineal care independently. This is consistent with an Indian study among caretakers of adolescents with ID, where 80.9% of girls were unable to manage menstruation on their own, and caregivers reported significant practical and emotional burden in supporting menstrual hygiene (2, 3). A significant association was observed between level of intellectual disability and hygiene dependence, with adolescents with moderate intellectual disability demonstrating higher dependence compared to those with mild intellectual disability. This reflects greater functional limitations and increased caregiving needs in this group. While this dependence reflects functional limitations, it also highlights the central role of caregivers in ensuring safe and dignified menstrual practices. From a salutogenic lens, caregiver involvement can be viewed as a key general resistance resource, enabling continuity of care, supervision, and emotional reassurance. Strengthening caregiver capacity through targeted education and training may help transition from complete dependence to supported self-care over time. In addition, educational sessions conducted for school authorities during the study further emphasized the importance of institutional support. Improving access to clean, private, and menstrual friendly sanitation facilities within schools may play a critical role in reducing functional limitations and improving school participation.

The findings from recent Indian and global reviews on menstrual health among persons with disability also describe compromised menstrual hygiene due to limited privacy, inaccessible toilets, low awareness, and lack of tailored support, stressing that safe menstrual hygiene depends heavily on caregiver training and institutional support (5, 25, 26). Menstrual-related challenges such as heavy menstrual flow, soiling accidents, and school absenteeism were commonly reported in the present study, reflecting the substantial functional impact of menstruation on daily life among adolescents with intellectual disability. These findings are consistent with evidence from low- and middle-income settings, where adolescents with disabilities often encounter multiple barriers, including inadequate sanitation infrastructure, lack of privacy, and limited access to appropriate support systems (3, 27). In addition to these contextual constraints, the presence of underlying symptoms such as pain, irregular cycles, and behavioral changes further compounds these challenges, leading to increased dependence and disruption of routine activities, including school participation. Therefore, menstrual health challenges in this population arise from an interplay of both symptom-related factors and environmental or contextual limitations, rather than from biological factors alone. Together, these results highlight that menstrual health for adolescents with ID cannot be addressed through products alone; it requires capacity-building of caregivers and schools to provide consistent, respectful and dignified support.

### 4.2 Premenstrual symptoms and multidomain clusters

Premenstrual symptoms were observed across psychological, physical, and behavioral domains, with considerable overlap between domains. Exploratory inferential analysis did not demonstrate statistically significant differences in psychological or physical domain scores between adolescents with mild and moderate intellectual disability. Behavioral domain scores were relatively higher among adolescents with mild intellectual disability, with a trend toward statistical significance. This pattern may reflect a greater ability to express behavioral and emotional changes among individuals with relatively higher functional capacity. These patterns are consistent with earlier work on PMS in adolescents with intellectual disability, where mood swings, irritability and behavioral dysregulation are reported as prominent features. Ibralic et al. (8) observed that adolescent girls with ID experience a range of PMS symptoms similar to their neurotypical peers, but functional impact may be greater due to communication and adaptive limitations. The strong overlap across domains highlights the multidimensional nature of premenstrual symptoms in this population.

In our study, majority of the girls met criteria for the pain-related cluster, with dysmenorrhea reported by 94.2% of adolescent girls. Behavioral symptoms were also highly prevalent, with nearly nine out of 10 girls exhibiting moderate to severe functional impairment, particularly in the form of premenstrual irritability, aggression, and emotional dysregulation. Psychological symptoms were common as well, with approximately 70%−80% of participants reporting moderate to severe mood variability, anxiety, or irritability. This strong overlap echoes broader disability literature showing that adolescents with disabilities are more likely than their peers to have multiple concurrent menstrual problems, including pain, behavioral disturbance and hygiene difficulties (6, 23). This clustering of somatic and behavioral symptoms is consistent with broader disability literature, which indicates that menstruation may exacerbate pre-existing behavioral issues, particularly in those with limited ability to verbalize discomfort or seek help (9). Despite the high symptom burden, the identification of cyclical behavioral and emotional changes provides an opportunity for anticipatory care. Caregiver awareness of these patterns can serve as a coping resource, allowing timely behavioral support, environmental modification, and structured routines. Such anticipatory strategies may reduce the severity of functional impairment and improve overall wellbeing.

### 4.3 Multidisciplinary care and supportive strategies

Our pattern of multidisciplinary recommendations is congruent with clinical guidance documents that argue girls with ID should be offered the same menstrual management options as other adolescents, but with additional attention to communication, consent and caregiver involvement (23, 28) The emphasis in our study on non-pharmacological methods (hot fomentation, hydration, local measures), careful use of analgesics and iron–folic acid supplementation, and targeted referrals for suspected PCOS or endocrine causes is consistent with recommended stepwise management (7, 24). Similarly, our psychiatry recommendations on caregiver-mediated behavioral strategies, such as structured routines, sensory calming techniques, and reinforcement approaches, were widely recommended. These strategies can be interpreted as salutogenic resources that enhance predictability, emotional regulation, and adaptive functioning. The integration of such approaches into routine care may improve both adolescent outcomes and caregiver confidence (9, 29).

### 4.4 Yoga-based practices in a supportive care context

A distinctive aspect of this study is the inclusion of yoga-based recommendations as part of routine supportive care. Evidence from general adolescent populations suggests that yoga practices may reduce dysmenorrhea, improve emotional regulation, and enhance overall wellbeing (15, 16). A recent trial among school girls reported significant reductions in pain intensity and duration of dysmenorrhea following a structured yoga programme, with associated improvements in absenteeism and wellbeing (17). While most of these studies have been conducted in neurotypical adolescents, the basic mechanisms such as modulation of autonomic balance, reduction of muscle spasm, and improved coping with pain and stress, are biologically plausible for girls with ID as well. Research on meditation and yoga for primary dysmenorrhea is ongoing and suggests that such non-pharmacological interventions may be a valuable adjunct in adolescent reproductive health (30, 31). Our findings support the feasibility of tailoring simple yoga modules (breathing, relaxation, pain-relief postures, asanas and chanting) to the cognitive and functional level of adolescents with ID, with active involvement of caregivers and teachers. In addition to potential symptom relief, yoga-based practices may function as a salutogenic resource by promoting body awareness, relaxation, and self-regulation. These practices, when adapted to the cognitive and functional abilities of adolescents with intellectual disability, may enhance coping capacity and provide a structured, culturally acceptable form of supportive care.

Taken together, the present study and existing literature point toward several public-health imperatives. First, routine menstrual and PMS screening should be integrated into special-school health services, with simple tools that combine symptom and functional assessment. Second, caregiver and teacher training is critical, as safe menstrual hygiene and behavioral support for adolescents with ID depend heavily on informed and confident adults. Third, yoga and other low-cost body–mind interventions can be explored as scalable components of adolescent-friendly health services in disability settings, especially within programmes such as RBSK and RMNCH+A in India.

### 4.5 Salutogenic interpretation of findings

While the study primarily documents menstrual related challenges, these findings can be reinterpreted through a salutogenic lens by identifying existing coping resources within caregiving and institutional environments. Caregiver involvement, structured routines, behavioral strategies, and school level support systems can be conceptualized as general resistance resources that facilitate coping with menstrual challenges in adolescents with intellectual disability.

These resources contribute to improved predictability, assistance, and emotional reassurance, which are essential for coping in this population. Although formal constructs such as sense of coherence were not directly measured in this study, the presence of structured caregiving and supportive environments suggests potential pathways to enhance comprehensibility, manageability, and meaningfulness of menstrual experiences. Thus, the findings highlight not only the burden of symptoms but also the role of supportive systems in shaping health experiences, reinforcing the relevance of a salutogenic perspective in understanding menstrual health in adolescents with intellectual disability.

### 4.6 Public health implications

The findings of this study have several implications for public health practice. Routine screening for menstrual and premenstrual symptoms should be integrated into special school health services. Capacity building of caregivers and teachers is essential to ensure appropriate support for menstrual hygiene and behavioral challenges. Additionally, low cost and culturally acceptable approaches such as yoga may be explored as complementary strategies within existing programmes such as Rashtriya Bal Swasthya Karyakram and RMNCH plus A.

### 4.7 Strengths and limitations

This study focuses on a vulnerable and under researched population and adopts a multidisciplinary assessment approach integrating gynecological, psychiatric, and supportive care perspectives. The use of structured tools and a school-based setting enhance the practical relevance of the findings. Exploratory analysis further provides insight into associations between level of intellectual disability and menstrual health variables. However, certain limitations should be acknowledged. The sample size was modest and restricted to two special schools in a single geographic area, which may limit generalisability. Data were based primarily on caregiver reports, which may introduce proxy reporting bias, including potential overestimation of observable behaviors and underreporting of subjective symptoms. Although behavioral assessments were conducted by multidisciplinary team, formal triangulation using structured observational tools or adolescent self-report measures was not undertaken. The cross-sectional design limits causal inference, and salutogenic constructs such as sense of coherence were not directly measured. The findings should therefore be interpreted as exploratory and context specific.

## 5 Conclusion

Adolescent girls with intellectual disability experience a high burden of menstrual and premenstrual challenges across physical, psychological, and behavioral domains, leading to significant functional limitations and caregiver dependence. The findings highlight that these challenges are multidimensional and often influenced by both symptom-related and contextual factors. Within a salutogenic perspective, existing caregiving and institutional supports may act as important resources that facilitate coping and functional adaptation. Strengthening these supports, along with incorporating structured screening and caregiver-focused education, may improve menstrual health management in this population. The study provides context-specific, exploratory evidence to inform future research. Larger, longitudinal, and interventional studies are required to evaluate the effectiveness of multidisciplinary approaches, including behavioral strategies and yoga-based practices, in improving outcomes among adolescents with intellectual disability.

## Data Availability

The raw data supporting the conclusions of this article will be made available by the authors, without undue reservation.
